# The Genetic Causal Effect of Autoimmune Diseases on pan-Cancers: Evidence from Mendelian Randomization

**DOI:** 10.7150/jca.103693

**Published:** 2025-01-01

**Authors:** Yubin Long, Yujun Pan, Houzhi Yang, Xiangbin Wang, Zichao Jiang, Tianwei Sun

**Affiliations:** 1 Tianjin Medical University, Tianjin, China.; 2 Department of Spinal Surgery, Tianjin Union Medical Center, Tianjin, China.; 3 Department of Spinal Surgery, The Central Hospital of Shaoyang, Shaoyang, Hunan, China.; 4 Department of Spinal Surgery, Affiliated Hospital of Chengde Medical University, Chengde, Hebei, China.; 5 Department of Orthopedic Surgery, Hunan Engineering Research Center of Biomedical Metal and Ceramic Implants, National Clinical Research Center for Geriatric Disorders, Xiangya Hospital, Central South University, Changsha, China.

**Keywords:** autoimmune diseases, pan-cancers, mendelian randomization, causal analysis

## Abstract

**Background:** Whether autoimmune diseases caused any effects on the risk of cancers remained yet clarified. This study aimed to investigate the causal effect of autoimmune diseases on pan-cancers through mendelian randomization (MR) analysis.

**Method:** The GWAS summary datasets of 10 autoimmune diseases were derived from the IEU or UK biobank website. The GWAS summary datasets of 39 cancers were derived from the FinnGen website. The Inverse-Variance Weighted (IVW) method was used to analyze the causal association between auto-immune diseases and pan-cancers.

**Results:** MR analysis indicated that inflammatory bowel disease would significantly increase the risk of rectum cancer, basal cell carcinoma and skin cancer, while Crohn's disease only showed causal association with increased risk of skin cancer. Rheumatoid arthritis significantly increased the risk of thyroid gland cancer, while reduce the risk of overall cancers and 7 other types of cancer outcomes. Ankylosing spondylitis showed causal relationship with increased risk of overall cancers and 5 other types of cancer outcomes. Multiple sclerosis significantly increased the risk of overall cancers and 5 other types of cancer outcomes, while reduce the risk of pancreas cancer (P=0.04). Parkinson's disease significantly reduced the risk of rectum cancer and skin cancer. Systemic lupus erythematosus significantly increased the risk of 3 types of cancer, while reduced the risk of kidney and urinary tract cancer. Psoriasis significantly increased the risk of head neck and oral cavity cancer, while reduced the risk of 3 other types of cancer.

**Conclusion:** This study provides novel genetic epidemiological evidence linking several autoimmune diseases to various cancer, both retrospective studies and basic research on this topic should carefully consider the genetic susceptibility to autoimmune diseases in relation to cancer, including potential risk and protective factors. These findings may inform preventive strategies and treatment approaches.

## Introduction

Autoimmune diseases are a category of systemic immunological dysfunction disorders, driven by a prolonged and abnormal adaptive immune response directed against self-antigens 1. There were approximately 5% of the global population is afflicted with autoimmune diseases 2. Among various autoimmune diseases, although their pathological mechanisms differ, they are predominantly characterized by an abnormal and prolonged adaptive immune response. Which primarily mediated by heightened B cell and T cell activities, a loss of immune tolerance toward self-antigens and leading to an imbalance in inflammatory cytokines 3. For example, in systemic lupus erythematosus (SLE), there is abnormal B cell activation that results in the overproduction of anti-nuclear antibodies (ANAs). These ANAs form complexes with nuclear components, which then deposit in various tissues and organs, accompanied by an increased level of inflammatory cytokines, especially IL-6 and TNF-α. Which ultimately triggers an inflammatory response and leads to tissue damage 4. A significant feature of ankylosing spondylitis (AS) is the abnormal activation of Th1 and Th17 cells, which produce pro-inflammatory chemokines such as IFN-γ and IL-17. These cytokines play a significant role in influencing disease progression through various immunological mechanisms 5. In rheumatoid arthritis (RA), both Th1 and Th17 cells, along with B cells, are aberrantly activated, and the function of T Regulatory cells (Tregs) is compromised, ultimately failing to maintain immune tolerance 6.

These immune characteristics may mediate the growth, invasion and metastasis of tumors through interactions and signal transduction pathways, playing a crucial regulatory role in the occurrence and development of tumor 7. The immune cells that play a major anticancer role in the tumor microenvironment primarily consist of cytotoxic T lymphocytes (CTLs) and helper T cells. The CD8^+^ CTLs could promote their anticancer effects by releasing IFN-γ and TNF-α, which trigger cytotoxic responses in cancer cells 8. While Tregs in the tumor microenvironment could suppress immune response by secreting cytokines such as IL-10 and TGF-β, thereby protecting tumor cells from attack. Myeloid-derived suppressor cells (MDSCs) could inhibit the activity of CD8+ cytotoxic T cells, allowing tumor cells to evade the surveillance of the immune system through various mechanisms. Which ultimately promotes tumor growth and metastasis 9. Considering the close relationship between tumors and the immune system, cancer immunotherapy, particularly focusing on immune checkpoints such as PD-L1 is recognized as one of the most promising areas in clinical oncology research. 10.

There are various of intricate connection between autoimmune diseases and cancer, both of which are influenced by the immune system and involve immune cells and cytokines such as TNF-α and IL-6 11, 12. Their connection is also evidenced by the application of antibody therapeutics and immunoregulation in clinical settings. For example, the anti-CD20 antibody has shown effectiveness in the treatment of both lymphoid malignancies and autoimmune disorders 13. Conversely, although anti-TNF antibody therapies have transformed the treatment of autoimmune diseases, changes in immune status caused by these TNF antibodies may unintentionally facilitate the development and progression of cancer 14. The impact of immunotherapeutic agents targeting specific genes, such as anti-IL-7R 13, anti-IL-6 15 and immune checkpoint inhibitors 16, their effect on cancer and autoimmune diseases is receiving extensive scrutiny. Eitan Giat *et al.* conducted a systematic review summarizing the potential associations between cancer and autoimmune disorders, highlighting significant links found in previous studies between RA, SLE and various cancers 17. Given that autoimmune diseases involve dysregulation of the innate immune system, we propose that this impaired immune response may promote oncogenesis. Therefore, identifying specific causal relationships between autoimmune diseases and different tumor types is essential. Such insights could clarify the immunological mechanisms underlying both autoimmune diseases and cancer, with important implications for their clinical management and prognosis. Considering the inherent immunological connections and the additional link provided by immunotherapy, traditional meta-analyses and reviews 18 may not yield valid and authentic conclusions.

Mendelian randomization (MR) is a methodological approach that employs measurable genetic variations to investigate the causal effects of an exposure on an outcome 19. By leveraging the random distribution of genetic variants, MR strengthens causal inference, addresses the limitations inherent in observational studies, and provides valuable insights into the etiology of diseases that are difficult to elucidate using other epidemiological techniques 20. It effectively reduces confounding and addresses reverse causation compared to observational studies 21 providing evidence for causality in scenarios where randomized trials are either infeasible or unethical 22. In our study, as detailed in Table 1.

## Methods and data

### Study design

**Figure 1:**
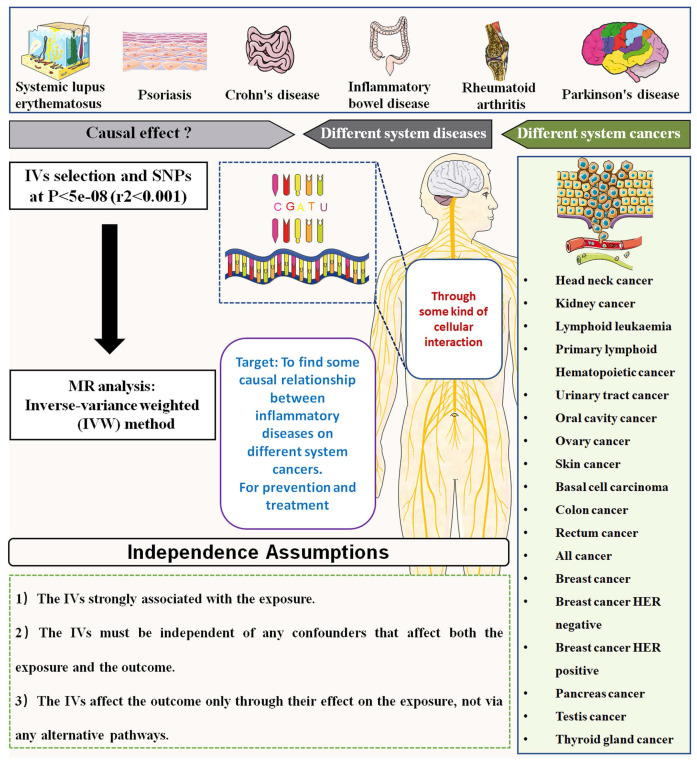
The flow gram of mendelian randomization analysis between autoimmune diseases and the risk of various cancers.

This study aimed to investigate the causal association between autoimmune diseases and risk of pan-cancers. The autoimmune diseases included SLE, Psoriasis, Crohn's disease, inflammatory bowel disease (IBD), Ulcerative colitis (UC), Alzheimer's disease (AD), RA, Parkinson's disease (PD), AS and multiple sclerosis (MS). The outcomes of cancer included 39 types of cancers, which was mainly classified according to the occurrence site. The mendelian randomization analysis using the inverse variance weighted (IVW) method was applied to analyze the causal association between autoimmune diseases and pan-cancers. A brief schematic flowchart outlining the analysis process is presented in **Figure 1**.

### Source of GWAS summary dataset

In this study, the genome-wide association study (GWAS) summary datasets of the autoimmune diseases were derived from prior GWAS investigations that reported the largest sample sizes for these autoimmune diseases. Detailed information regarding these autoimmune diseases can be found in Table 1. The GWAS summary datasets for pan-cancers were obtained from the FinnGen database (https://www.finngen.fi/en/access_results), encompassing 39 cancer types located in various anatomical sites, with cancer case numbers ranging from 197 to 87,531. Comprehensive details for the GWAS summary datasets associated with these 39 cancers are presented in Table S1. To minimize potential ethnic stratification bias, our analysis was confined to European population.

### Mendelian randomization analysis

To evaluate the causal relationships between autoimmune diseases and pan-cancers, we applied the IVW method, enhanced with multiplicative random effects based on the number of instrumental variants. Single nucleotide polymorphisms (SNPs) were extracted from the GWAS summary data concerning the exposure to serve as genetic instruments for autoimmune diseases. Three critical assumptions must be fulfilled during the MR analysis process:

SNP-Exposure Association: The genetic instruments must have a significant association with the exposure, requiring SNPs to display strong correlations with a p-value of less than 5e-8.

SNP-Outcome Independence: The chosen SNPs should not show a strong association with the pan-cancer outcomes, necessitating p-values greater than 5e-5 for these cancer outcomes.

Exclusivity of Effect: It is essential that the genetic instruments influence the outcomes solely through their effect on the exposure, ensuring that the SNPs affect pan-cancers exclusively via autoimmune diseases.

To adhere to these principles, we implemented several SNP selection strategies:

Linkage Disequilibrium Exclusion: We employed an LD clustering algorithm, setting parameters to P≤5e-8 and r²=0.001 to eliminate SNPs that might introduce bias due to linkage disequilibrium, thus minimizing complex LD effects.

Gene Window Restriction: We limited the genomic windows of SNPs to 100 kb to enhance analytical precision.

Pleiotropy Mitigation: To reduce the risk of pleiotropy, which could distort our results, we excluded any SNPs connected to the exposure if five or more were involved.

Strength Evaluation: The strength of the association between the instrumental variables (IVs) and both autoimmune and pan-cancer traits was assessed using the F-value. We considered a standard F-value exceeding 20 as indicative of robust associations, ensuring the efficacy of our genetic instruments. The F-value was calculated using the formula:

*F*=*R*^2^(*n*-1-*k*)/(1-*R*^2^)*k*

Where *R*^2^ denotes instrumental variance, *n* is the sample size, and *k* represents the number of IVs.

In cases where only a single SNP was identified, we utilized the Wald ratio method to estimate the causal effect for that specific scenario. For situations where the number of SNPs ranged from 1 to 3, we employed a fixed effect model for data analysis. Conversely, when 3 or more SNPs were selected, we implemented a random effects model in conjunction with the IVW method for MR analysis. The odds ratio (OR) and 95% confidence intervals (CI) were calculated to evaluate the strength and precision of the causal associations, with a p-value of less than 0.05 signifying statistical significance.

## Results

This study applied the IVW method to investigate the causal association between autoimmune diseases and pan-cancers. As shown in table 1, there were 10 autoimmune diseases included for analysis, comprising SLE, psoriasis, IBD, CD, UC, AD, PD, MS, RA and AS. The outcomes in this study included 39 cancers of different occurrence sites, the detailed information was summarized in the Table S1.

**Figure 2:**
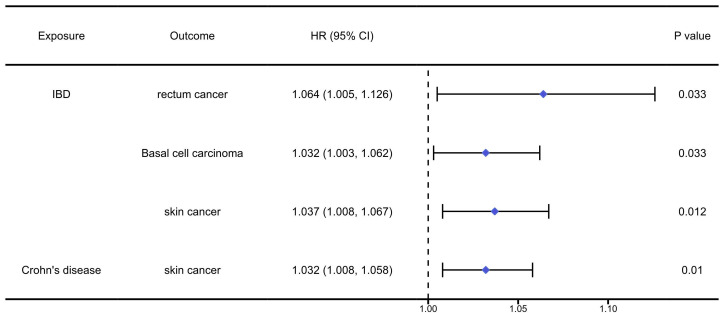
The positively causal association between autoimmune diseases of digestive system and cancers identified by the IVW method. IBD: inflammatory bowel disease.

The analysis results of the causal association between digestive autoimmune diseases and pan-cancers were presented in table S2-4 and Figure 2. The analysis results indicated that IBD would significantly increase the risk of rectum cancer (OR=1.064, 95%CI: 1.005, 1.126, P=0.033), basal cell carcinoma (OR=1.032, 95%CI: 1.003, 1.062, P=0.033), and skin cancer (OR=1.037, 95%CI: 1.008, 1.067, P=0.012). Meanwhile, we found that Crohn's disease would increase the risk of skin cancer (OR=1.032, 95%CI: 1.008, 1.058, P=0.01).

**Figure 3:**
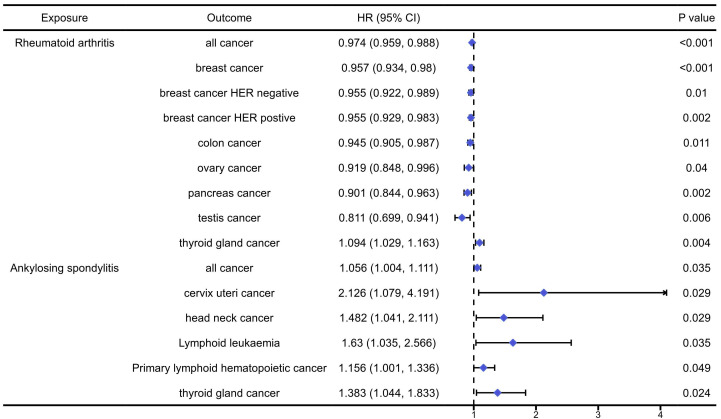
The positively causal association between autoimmune diseases of musculoskeletal system and cancers identified by the IVW method.

The analysis results of the causal association between musculoskeletal autoimmune diseases and pan-cancers were presented in table S5-6 and Figure 3. The analysis results indicated that RA would significantly increase the risk of thyroid gland cancer (OR=1.094, 95%CI: 1.029, 1.162, P=0.004), while reduce the risk of overall cancers (OR=0.919, 95%CI: 0.848, 0.996, P<0.001), breast cancer (OR=0.957, 95%CI: 0.934, 0.980, P<0.001), HER negative breast cancer (OR=0.955, 95%CI: 0.922, 0.989, P=0.01), HER positive breast cancer (OR=0.955, 95%CI: 0.929, 0.983, P=0.002), colon cancer (OR=0.945, 95%CI: 0.905, 0.987, P=0.011), ovary cancer (OR=0.919, 95%CI: 0.848, 0.996, P=0.04), pancreas cancer (OR=0.901, 95%CI: 0.844, 0.963, P=0.002) and testis cancer (OR=0.811, 95%CI: 0.699, 0.941, P=0.006). In addition, we found that AS could significantly increase the risk of overall cancers (OR=1.056, 95%CI: 1.004, 1.111, P=0.035), cervix uteri cancer (OR=2.126, 95%CI: 1.004, 1.111, P=0.029), head neck cancer (OR=1.482, 95%CI: 1.041, 2.111, P=0.029), lymphoid leukaemia (OR=1.630, 95%CI: 1.035, 2.566, P=0.035), primary lymphoid hematopoistic cancer (OR=1.156, 95%CI: 1.001, 1.336, P=0.049) and thyroid gland cancer (OR=1.383, 95CI: 1.044, 1.833, P=0.024).

**Figure 4:**
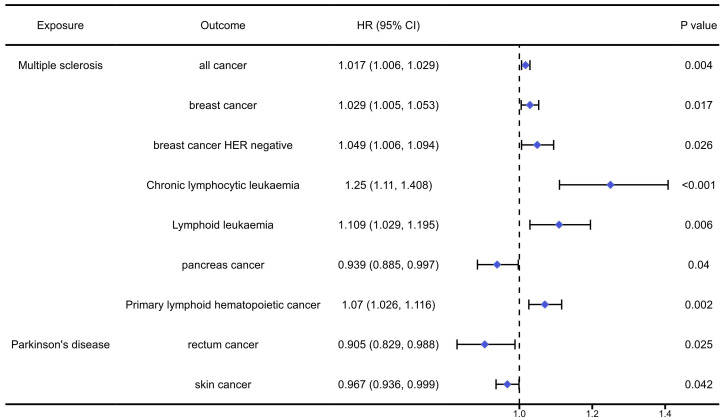
The positively causal association between autoimmune diseases of nervous system and cancers identified by the IVW method.

The analysis results of the causal association between nervous autoimmune diseases and pan-cancers were presented in table S7-9 and Figure 4. The analysis results indicated that MS could significantly increase the risk of overall cancers (OR=1.017, 95%CI: 1.006, 1.029, P=0.004), breast cancer (OR=1.029, 95%CI:1.005, 1.053, P=0.017), HER negative breast cancer (OR=1.049, 95%CI: 1.006, 1.094, P=0.026), chronic lymphocytic leukaemia (OR=1.250, 95%CI: 1.110, 1.408, P<0.001), lymphoid leukaemia (OR=1.109, 95%CI: 1.029, 1.195, P=0.06), primary lymphoid hematopoietic cancer (OR=1.070, 95%CI: 1.026, 1.116, P=0.02), while reduce the risk of pancreas cancer (OR=0.939, 95%CI: 0.885, 0.997, P=0.04). In addition, the PD would significantly reduce the risk of rectum cancer (OR=0.908, 95%CI: 0.829, 0.988, P=0.025) and skin cancer (OR=0.967, 95%CI: 0.936, 0.999, P=0.042).

**Figure 5:**
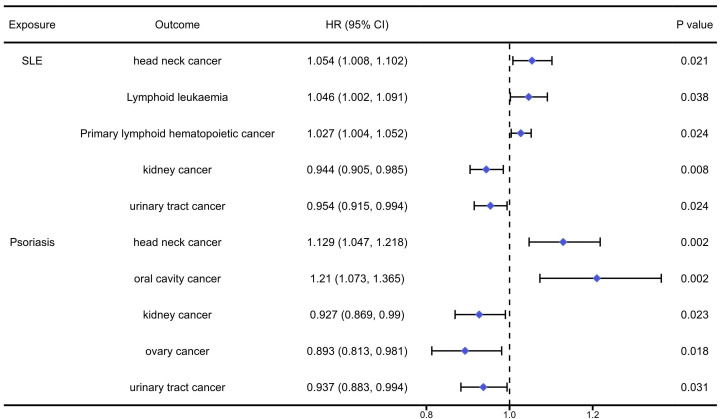
The positively causal association between systemic autoimmune diseases and cancers identified by the IVW method.

The analysis results of the causal association between systemic autoimmune diseases and pan-cancers were presented in table S10-11 and Figure 5. We found that SLE would significantly increase the risk of head neck cancer (OR=1.054, 95%CI: 1.008, 1.102, P=0.021), primary lymphoid hematopoietic cancer (OR=1.027, 95%CI: 1.003, 1.051, P=0.038) and lymphoid leukaemia (OR=1.046, 95%CI: 1.002, 1.091, P=0.024), while reduced the risk of kidney cancer (OR=0.944, 95%CI: 0.905, 0.985, P=0.008) and urinary tract cancer (OR=0.954, 95%CI: 0.915, 0.994, P=0.024). Meanwhile, we found that Psoriasis would significantly increase the risk of head neck cancer (OR=1.129, 95%CI: 1.047, 1.218, P=0.002) and oral cavity cancer (OR=1.210, 95%CI: 1.073, 1.365, P=0.002), while it could reduce the risk of kidney cancer (OR=0.927, 95%CI: 0.869, 0.990, P=0.023), ovary cancer (OR=0.893, 95%CI: 0.813, 0.981, P=0.018) and urinary tract cancer (OR=0.937, 95%CI: 0.883, 0.994, P=0.031).

## Discussion

Autoimmune diseases are conditions in which the immune system erroneously targets and harms healthy tissues and organs of the body 23. There appears to be a complex relationship between autoimmune disorders and cancer. Although there were growing recognition of the significant role that immune imbalance plays in tumor development, metastasis and the tumor microenvironment, it remains unclear whether the incidence of specific immune diseases increases the risk of cancer. In this study, we conducted a MR analysis to investigate the causal effect of major autoimmune diseases on 39-type cancers.

Key findings including the heightened risk of overall cancer in patients with AS (OR=1.056, 95%CI: 1.004, 1.111, P=0.035) and MS (OR=1.017, 95%CI: 1.006, 1.029, P=0.004). Notably, Crohn's disease showed no causal relationship with risk of rectum cancer, while it could increase the risk of skin cancer. RA was found to have a paradoxical relationship, showing both an increased risk for thyroid gland cancer while simultaneously reducing the risk of overall cancer and several cancers. Similar contrasting patterns were observed in AS, SLE and Psoriasis, indicating the multifaceted roles these conditions play in cancer genetic epidemiology. The findings emphasize the necessity for personalized monitoring and intervention strategies tailored to individuals with autoimmune diseases. These results highlight the complex interplay between autoimmune diseases and cancer risk, providing valuable insights for further research and clinical considerations.

### Inflammatory bowel disease (IBD) and Crohn's disease

The occurrence and development of IBD are related to immune abnormalities, genetic and environmental factors, and patients may experience server symptoms. Research indicated that IBD patients have an increased risk of developing cancer, potentially due to immunosuppression 24. We found that IBD patients have a 6.4% higher risk of rectum cancer compared to the general population, with a 95% CI of 1.005-1.126 and a P-value of 0.033. More recently, patients with IBD have also been shown to be at increased risk of developing extra-intestinal malignancies 25. Beaugerie *et al.* also pointed out that the risk of cholangiocarcinoma in IBD was 2-6 times higher than that of the general population 26. Consistent with these findings, we also found that IBD can improve the incidence rate of basal cell carcinoma (OR=1.032, 95% CI: 1.003, 1.062, P=0.033) and skin cancer (OR=1.037, 95% CI: 1.008, 1.067, P=0.012). Whether Crohn's disease increases the risk of colorectal cancer has long been a contentious issue. Jess *et al.* conducted a meta-analysis indicating that patients with Crohn's disease have an overall increased risk of developing small intestinal cancer 27. While Liu *et al.* conducted a MR analysis found that there were no significant genetic association between Crohn's disease and various cancers, including gastrointestinal cancer 28. In our study, we found that Crohn's disease showed strongly causal effect in increased risk of skin cancer, while not in gastrointestinal.

### Rheumatoid arthritis (RA)

RA is a chronic autoimmune inflammatory disease targeting joints, which may be associated with cancer risk in addition to joint destruction and inflammation 29. We found that RA can reduce the risk of all cancer, breast cancer, colon cancer, ovary cancer, pancreas cancer and testis cancer, while increase the risk of thyroid gland cancer. Consistent with our results, Beydon et.al found that the risk of breast cancer with standardized incidence ratio (SIR) of 0.91 and pancreatic cancer with SIR of 0.90 was lower in RA patients compared to the general population 30. Similarly, Simon et.al reported that patients with RA had a decreased risk of colon or colorectal cancer, with SIR ranging from 0.49 (0.26-0.83) to 0.96 (0.56-1.54) 31. In addition, Cui et.al reported that male RA patients exhibited 4.90-fold higher odds (95% CI: 1.49, 16.07) for thyroid cancer compared to controls 32. However, in a study investigating the risk of ovarian malignancy in women with RA, the authors found no significant association between RA and ovarian cancer risk 33.

### Ankylosing spondylitis (AS)

AS is a type of inflammatory arthritis that primarily affects the spine and sacroiliac joints (where the spine connects to the pelvis) 34. We found that AS can increase the risk of all cancer, cervix uteri cancer, head neck cancer, lymphoid leukemia, primary lymphoid hematopoietic cancer and thyroid gland cancer (OR=1.383, 95% CI: 1.044, 1.833, P=0.024). Zhang et.al reported that an increased overall cancer risk in patients with AS compared to the general population 35. According to a retrospective cohort study data from patients with AS and patients without AS collected between 2000 and 2008 from the Taiwanese National Health Insurance Research Database (NHIRD), male AS patients had a significantly increased risk of developing bone cancer (SIR: 6.00, 95% CI: 1.23, 17.5) and prostate cancer (SIR: 1.64, 95% CI: 1.04, 2.47) compared to non-AS patients. Female AS patients had a higher risk of developing colon cancer (SIR: 1.73, 95% CI: 1.19, 2.45) compared to those without AS. It reported that AS patients also had an elevated risk of hematological malignancies like lymphoma (SIR: 2.10, 95% CI: 1.32, 3.19) 35. Furthermore, a MR study also indicated that genetically determined AS was causally correlated with a remarkably increased risk of lung cancer among European populations 36.

### Multiple sclerosis (MS)

MS is a chronic autoimmune inflammatory disease that affects the central nervous system (brain, spinal cord, and optic nerves) 37. According to our results, we found that MS can increase the risk of all cancer, breast cancer, chronic lymphocytic leulaemia (OR=1.250,95% CI: 1.110, 1.408, P <0.001), lymphoid leukemia and primary lymphoid hematopoietic cancer. However, MS can reduce the risk of pancreas cancer. Consistent with our results, Etemadifar *et al.* have reported an increased risk of certain cancers like breast cancer, urinary tract cancers, brain tumors, and lymphomas in MS patients 38. Furthermore, Liu et.al reported that MS patients exhibited enhanced pancreatic and ovarian cancer risk, and diminished breast and brain cancer risk 39.

### Parkinson's disease

Epidemiological studies have revealed that in most patients with PD, the incidence rate of various cancer types shows a downward trend. Numerous studies have shown an overall negative correlation between PD and various cancer risks, indicating that PD patients face lower cancer risks compared to the general population 40. Kim *et al.* reported that the risk of PD patients suffering from gastric cancer, thyroid cancer and hematological malignancies is relatively low 41. According to a study, PD can reduce the risk of rectal cancer with a hazard ratio (HR) of 0.905, a 95% CI of 0.829 to 0.988, and a P-value of 0.025. Additionally, PD also reduces the risk of skin cancer with a HR of 0.967, a 95% CI of 0.936 to 0.999, and a P-value of 0.042. Consistent with our results, a meta-analysis of 13 studies involving 343,226 PD patients showed that PD patients had a significantly decreased risk of colorectal cancer compared to those without PD (pooled risk ratio 0.79, 95% CI: 0.66-0.93), compared to Asian populations, this inverse association between PD and reduced colorectal cancer risk was more prominent in Western populations 42.

### Systemic lupus erythematosus (SLE)

SLE was found to be associated with higher cancer incidence rate and cancer related mortality; However, these findings are not entirely consistent 43. Meanwhile, SLE also increases the risk of lymphocytic leukemia and primary lymphocytic hematopoietic cancer, with corresponding OR of 1.046 and 1.027, 95% CIs of 1.002 to 1.091 and 1.004 to 1.052, and P of 0.038 and 0.024. On the contrary, SLE has been found to reduce the risk of kidney cancer and urinary tract cancer, with OR of 0.944 and 0.954, 95% CIs of 0.905 to 0.985 and 0.915 to 0.994, and P of 0.008 and 0.024. Consistent with this research result, Song *et al.* also reported that the risk of prostate cancer and skin melanoma 44. In a study involving 8751 SLE patients from Taiwan, Chang *et al.* found that the incidence rate of cancer increased, with the incidence rate ratio of 2.16 45. Apor *et al.* reported that the incidence rate of all hematological malignancies increased 2.9 times, with a 95% CI of 2.0 to 4.4 46. However, inconsistent with this research result, several cohort studies show that the risk of bladder cancer has increased, and its standardized incidence rate range is about 2.28, with a 95% CI of 1.62 to 3.12 47.

### Psoriasis

Psoriasis, as a chronic autoimmune disease, has attracted much attention for its association with malignant tumors. The latest authoritative research shows that the overall risk of cancer in psoriasis patients is significantly increased, especially for major diseases such as skin tumors and lymphoma. In our study, we found that psoriasis can increase the risk of head neck cancer (OR: 1.129, 95% CI: 1.047, 1.218, P =0.002) and oral cavity cancer (OR: 1.210, 95% CI: 1.073, 1.365, P =0.002). On the contrary, we found that psoriasis can reduce the risk of kidney cancer (OR:0.927, 95% CI: 0.869, 0.990, P =0.023), ovary cancer (OR: 0.893, 95% CI: 0.813, 0.981, P =0.018) and urinary tract cancer (OR: 0.937, 95% CI: 0.883, 0.994, P =0.031). Consistent with our results, a nationwide population-based study report in Taiwan, which found that psoriasis patients have a significantly increased risk of developing head and neck cancer, with an adjusted HR of 1.39 and a 95% CI of 1.18 to 1.64 48. Furthermore, the risk was higher in patients with severe psoriasis (adjusted HR 1.92, 95% CI: 1.41, 2.62) compared to those with mild psoriasis (adjusted HR 1.32, 95% CI: 1.10, 1.59) 49.

Overall, autoimmune diseases have genetically causal effect on the risk of various cancer. The mechanisms may include persistent abnormal activation of B cells and T cells, as well as impaired immune cell recognition of self-tissues. These factors can facilitate immune evasion by various tumor cells, thereby promoting tumor proliferation and survival. Additionally, autoimmune diseases lead to chronic inflammation, resulting in the secretion of various inflammatory factors such as IL-6, IL-10 and TGF-β, which play significant roles in tumor progression. Many studies suggested that immune responses in autoimmune diseases may play a crucial role in the carcinogenic process. However, there were also considerable studies observing that certain autoimmune diseases may be associated with a lower risk of cancer. It reflected the complexity of interactions between the immune system and genetic factors 17, 50. The immune cells and inflammatory responses were in a state of dynamic balance, it important to that the immune sensitivity of different cancer types, as well as their reactions to specific inflammatory factors, can vary significantly. It may result in opposing effects on the risk of different cancers associated with the same autoimmune disease. Therefore, it is essential to explore these connections in the context of specific autoimmune disease and cancer types.

### Strength and limitations

This study is the first to systematically investigate the causal association between autoimmune diseases and pan-cancers through MR analysis. Unlike observational studies, MR utilizes instrument variants to explore causal relationships, making it an appropriate method given the ethical constraints and challenges in data collection for clinical studies.

However, certain limitations must be acknowledged when generalizing the conclusions. Firstly, the causal association detected was limited to the European population, so caution is warranted when applying these findings to other populations. In this study, the cancers were classified based on their site of occurrence. While in clinical practice, cancers are often categorized using pathological typing and staging methods. Different pathological subtypes of cancer may exhibit significant differences in their pathogenesis and progression. Therefore, categorizing cancers solely by their site of occurrence in this study may not have identified some pathological types that are closely related to immune factors. Similarly, there is notable stratification by gender and age among patients with autoimmune diseases. This aspect needs to be further explored in future research. This aspect needs to be further addressed in future research. In addition, the IVW method does not allow us to determine whether the effect of autoimmune diseases on the risk of pan-cancers is direct or indirect. Further research should focus on addressing this issue.

## Conclusion

In summary, this study provides novel genetic epidemiological evidence that inherited lifetime exposure to specific autoimmune diseases is strongly associated with the risk of several types of cancer. These findings do not entirely align with previous retrospective or observational studies. Given the factors accompanying the diagnosis and treatment of autoimmune diseases, particularly the use of immunosuppressive treatments, both retrospective studies and basic research on this topic should carefully consider the genetic susceptibility to autoimmune diseases in relation to cancer, including potential risk and protective factors. These findings may help inform preventive strategies and treatment approaches.

## Supplementary Material

Supplementary tables.

## Figures and Tables

**Table 1:** the baseline data of GWAS summary dataset related to the autoimmune diseases.

Datasets	Source or PMID	Population	Case (n)	Control (n)
Systemic lupus erythematosus	26502338	European	5201	9066
Psoriasis	34927100	European	15967	28194
Inflammatory bowel disease	26192919	European	31,665	33,977
Crohn's disease	26192919	European	17,897	33,977
Ulcerative colitis	26192919	European	13,768	33,977
Alzheimer's disease	35379992	European	39,106	487,511
Parkinson's disease	IPDGC	European	33674	449056
Multiple sclerosis	31604244	European	47,429	68,374
Rheumatoid arthritis	36333501	European	22,350	74,823
Ankylosing spondylitis	23749187	European	9069	13578

IPDGC: International Parkinson's Disease Genomics Consortium

## Abbreviations

MR: Mendelian randomization
IVW: Inverse-Variance Weighted
SLE: systemic lupus erythematosus
ANAs: anti-nuclear antibodies
AS: ankylosing spondylitis
RA: rheumatoid arthritis
Tregs: T Regulatory cells
CTLs: cytotoxic T lymphocytes
MDSCs: Myeloid-derived suppressor cells
IBD: inflammatory bowel disease
UC: Ulcerative colitis
AD: Alzheimer's disease
PD: Parkinson's disease
MS: multiple sclerosis
GWAS: genome-wide association study
SNPs: Single nucleotide polymorphisms
IVs: instrumental variables
OR: odds ratio
CI: confidence intervals
